# Fundamentals of Monitoring Condensation and Frost/Ice Formation in Cold Environments Using Thin-Film Surface-Acoustic-Wave Technology

**DOI:** 10.1021/acsami.3c04854

**Published:** 2023-07-11

**Authors:** Xingchang Zeng, Huiling Ong, Luke Haworth, Yuchao Lu, Deyu Yang, Mohammad Rahmati, Qiang Wu, Hamdi Torun, James Martin, Xianghui Hou, Xianglian Lv, Weizheng Yuan, Yang He, Yongqing Fu

**Affiliations:** † Key Laboratory of Micro/Nano Systems for Aerospace, Ministry of Education and Shaanxi Key Laboratory of Micro and Nano Electromechanical Systems, School of Mechanical Engineering, Northwestern Polytechnical University, Xi’an 710072, P. R. China; ‡ Xi’an Institute of Applied Optics, Xi’an 710072, P. R. China; § Faculty of Engineering and Environment, Northumbria University, Newcastle upon Tyne NE1 8ST, U.K.; ∥ State Key Laboratory of Solidification Processing and Shaanxi Key Laboratory of Fiber Reinforced Light Composite Materials, Northwestern Polytechnical University, Xi’an 710072, P. R. China

**Keywords:** surface acoustic wave, frequency shift, condensation, icing monitoring, cold environments, multienvironmental dynamic factors

## Abstract

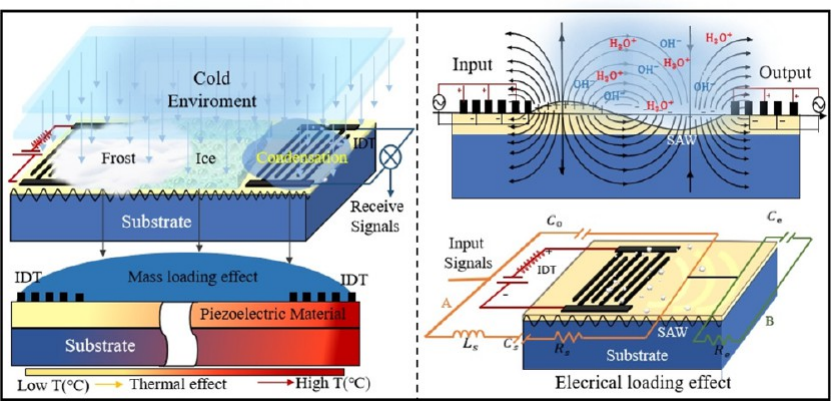

Moisture condensation, fogging, and frost or ice formation on structural surfaces cause severe hazards in many industrial components such as aircraft wings, electric power lines, and wind-turbine blades. Surface-acoustic-wave (SAW) technology, which is based on generating and monitoring acoustic waves propagating along structural surfaces, is one of the most promising techniques for monitoring, predicting, and also eliminating these hazards occurring on these surfaces in a cold environment. Monitoring condensation and frost/ice formation using SAW devices is challenging in practical scenarios including sleet, snow, cold rain, strong wind, and low pressure, and such a detection in various ambient conditions can be complex and requires consideration of various key influencing factors. Herein, the influences of various individual factors such as temperature, humidity, and water vapor pressure, as well as combined or multienvironmental dynamic factors, are investigated, all of which lead to either adsorption of water molecules, condensation, and/or frost/ice in a cold environment on the SAW devices. The influences of these parameters on the frequency shifts of the resonant SAW devices are systematically analyzed. Complemented with experimental studies and data from the literature, relationships among the frequency shifts and changes of temperature and other key factors influencing the dynamic phase transitions of water vapor on SAW devices are investigated to provide important guidance for icing detection and monitoring.

## 1 Introduction

Cold environments, especially those under high relative humidity (RH) levels, can lead to moisture condensation and the formation of frost, snow, or ice on the structural surfaces, imposing severe safety issues for aircraft wings,^1,2^ power transmission lines,^3−5^ and wind turbine blades.^6−8^ Mitigation strategies include measures on their prevention, elimination, and monitoring. However, sensing, monitoring, and/or removal of these hazardous formations in a cold environment are challenging due to the interplay of many environmental factors including temperature, RH, pressure, etc., which influence the phase transitions of water (among states of gas, liquid, and solid, including water vapor, fog, moisture, snow, frost, or ice).^9−11^ Frost or ice formation rates are also significantly increased with an increase of the RH values in air and affected by atmospheric pressure, wind speed, cold rains, and ice formation at different temperatures.^12,13^

Various techniques have been developed to monitor moisture condensation and the formation of frost, snow, and ice, among which ice monitoring is the most studied.^14−16^ Most techniques for monitoring ice/frost formation are based on in situ measurements of parameter changes for the properties of a specific sensor due to mass loading and changes of the dielectric constants, capacitance, inductance, optical properties, or vibrant frequencies (i.e., using an ultrasonic method or acoustic waves). These methods all have their merits and limitations,^15−32^ as presented in Table 1.

**Table 1:** Comparisons of In Situ Ice Measurement Techniques and Their Advantages and Limitations

detection technique	measurement principle	advantages	limitations
mechanical vibration	resonant frequency of the blade or sensor’s probe^18^ changes with ice accretion	sensitive to the presence and location of ice on any surface	insensitive to small-scale icing and susceptible to the various environmental factors, such as a loud noise, adsorption of water, and fog
		simple accessible and direct contact measurement	
microwave	impedance and electromagnetic energy of the waveguide change with ice accumulation on the waveguide surface^19,20^	sensitive to the presence and thickness of ice	susceptible to noises from the structure or environment
capacitive	capacitive value of the sensor’s element changes^21−23^ change with the dielectric substance, such as ice, snow, and water	sensitive to the presence and thickness of ice on any surface	limited area coverage based on discrete elements
		distributed measurement on the surface of the structure or embedded within the structure	effect of freezing temperatures on the performance of the sensing elements and low precision
impedance	impedance of the electrodes changes when ice approaches the electrodes, and then voltage is used to measure the changes^24^	sensitive to the presence and thickness of ice	great influence of an external attached disturbance on the sensitivity of the measuring unit
		distributed measurement on the surface of the structure or embedded within the structure	
optical	optical signal of reflection or the refractive index and scattering of the ice to show the ice presence, thickness, and type^25,26^	sensitive to the presence, type, thickness, and location of ice on both flat and bent surfaces	susceptible to dust
			complex in structure
			expensive equipment and difficult to measure the ice mass for thick layers
acoustic wave	velocity or frequency of propagating waves and amplitude changes when ice accumulates on the blade or sensor’s surface^27,28^	sensitive to the presence, type, thickness, mass, and location of ice on any surface	attenuation and propagation velocity affected by the piezoelectric substrate material
		distributed measurement on the surface of the structure or embedded within the structure.	
ultrasonic	ultrasonic waves’ amplitude and traveling time between the components change with variation of the ice physical variables^29^	sensitive to the presence, thickness, and location of ice on both flat and bent surfaces	negative effect of the mounting method on the measured structure and susceptible to water and surrounding noise^30^
IR thermography	electromagnetic wave signal changes form a map of temperature variations^31,32^	sensitive to the presence and location of ice on both flat and bent surfaces	only the presence of ice detectible and more susceptible to the ambient temperature, surface material, daylight, and weather conditions
			difficult to access and only applicable to a flat surface

As seen from Table 1, ultrasonic and acoustic wave technologies are two promising candidates to monitor ice/frost formation in a cold environment, and they are commonly based on monitoring of the vibration frequencies with the capability of ice thickness measurement.^15,33,34^ Another key advantage of these techniques is that they can also be used as active deicing or antiicing methods. For example, recent studies clearly show that, for ice mitigation, surface-acoustic-wave (SAW) devices can generate both acoustic wave vibrations and thermal effects on the device surface,^35,36^ thus offering great potential for both antiicing and deicing with a reasonably high efficiency.^37−40^ Therefore, it could be applied as one of the appropriate techniques for effectively tackling icing issues on structural surfaces. However, the conventionally used bulk piezoelectric ceramic-based ultrasonic or SAW devices have critical issues such as brittleness of the piezoelectric substrates (especially at high acoustic wave powers or large mechanical forces), rigidity, and noncompatibility with structural surfaces or microelectrics-based mass production technologies. On the contrary, thin-film-based SAWs have the advantage that they can integrate multiple functions into a single structure on different substrates, such as silicon, metals, glass, or polymers,^16^ and also flexible and wearable ones.^41^ This provides one of the best solutions to integrating thin-film SAW devices directly onto structural component surfaces (such as metal, glass, ceramic, polymer, or composites) for both monitoring and mitigation functions.

**Figure 1:**
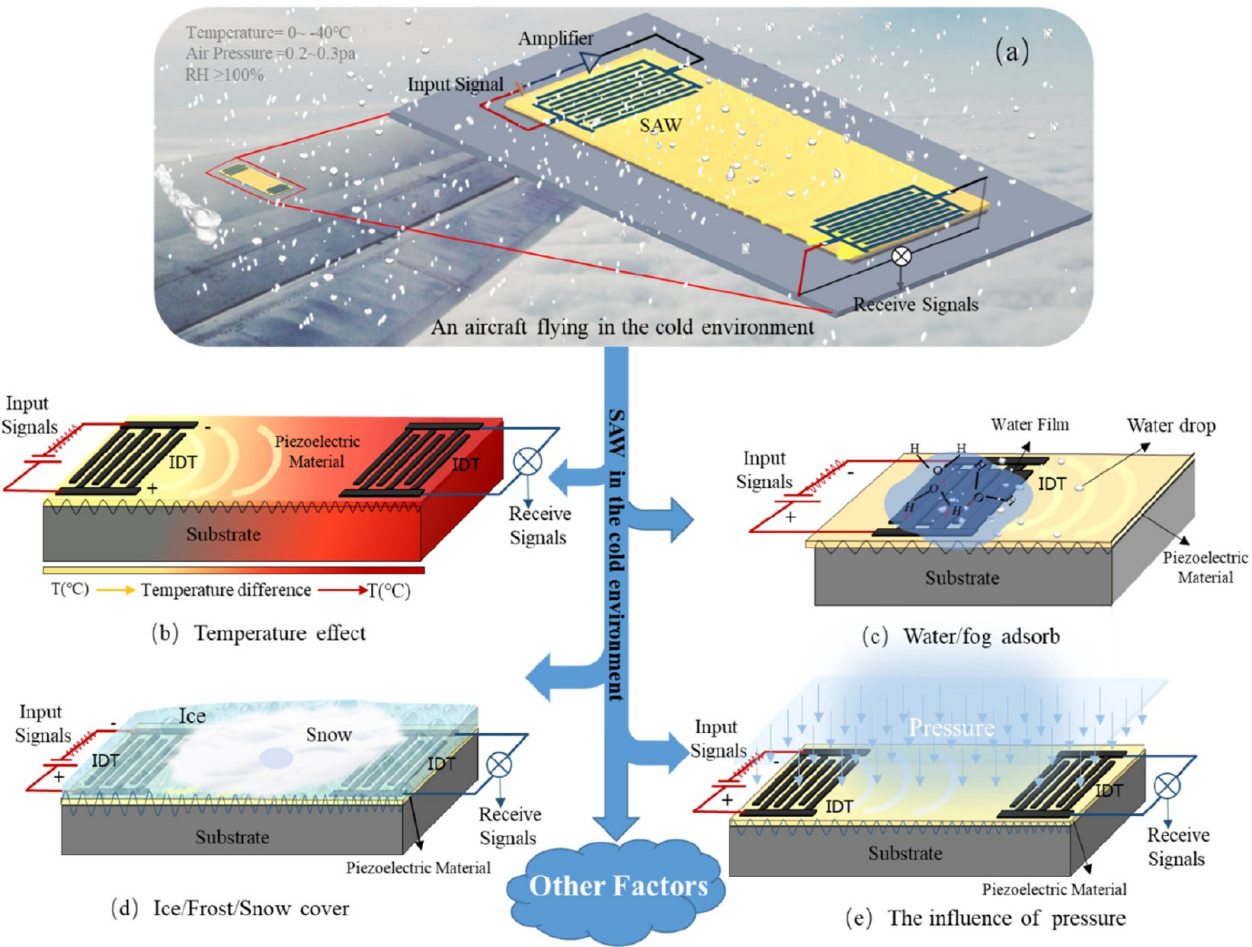
(a) Various phenomena that occur on the surface of a SAW sensor that is integrated with the structure of an aircraft in a cold environment. (b) Temperature effects due to the SAW device with temperature differences. (c) Schematic of water or fog adsorbed on the SAW device’s surface. (d) Schematic of ice/snow formed on the SAW device’s surface. (e) Schematic presentation of the pressure effect on the SAW device.

**Figure 2:**
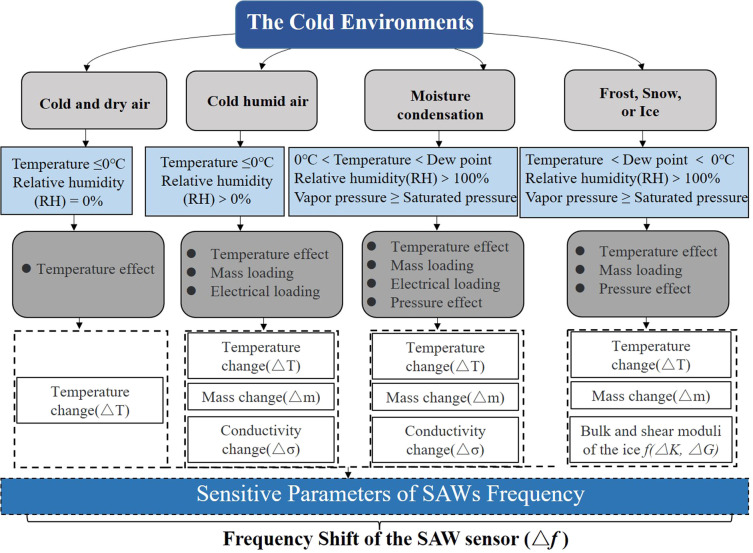
Key parameters of the frequency of the SAW sensor induced by a variety of environmental factors.

Various phenomena such as moisture condensation and water, fog, frost, snow, and ice formation will cause different changes in the physical or electrical properties (including temperature, mass loading, and electrical loading effects) of the SAW devices.^39^Figure 1 illustrates some commonly observed phenomena that occur on the surface of a standard SAW sensor, which could be integrated with an aircraft structure in a cold environment. Figure 2 summarizes the key parameters influencing frequency changes of the SAW sensor induced by a variety of environmental factors. Although the influences of each single parameter have been explored previously,^42−45^ the hybrid effects of multifactors in complex cold environments (which have truly existed in real application situations) have never been systematically studied in the literature.

In this paper, the key influences of various parameters, including temperature and/or humidity changes, and the formation of condensed moisture, fog, frost, or ice on SAW propagations in cold environments are systematically investigated based on our experimental studies and also those reported in the literature. The changes in the propagation velocity *v* of the SAWs induce changes in the resonant frequency *f*, i.e., *f* = *v*/λ, where λ is the SAW wavelength. In the cold environment, frequency shifts of the SAW device could be affected by a combination of temperature changes, electrical effects (e.g., conductivities or capacitance caused by the formation of moisture or other environmental factors), and mass loading effects (generated by the adsorption of water molecules or coverage of frost/ice at various temperatures) as well as changes in the RH values (Figure 2). To the best of our knowledge, this paper is the first systematic investigation considering these various key factors on the frequency changes of SAW devices in cold environmental conditions. The information is crucial for future designs of SAW sensors to predict different phenomena of moisture condensation and fog, snow, frost, or ice formation generated in cold environments.

## 2 Theoretical Analysis

The frequency shift (Δ*f*) of SAW sensors is affected by their physical, electrical, or electromagnetic properties due to mass loading, electrical loading, temperature, and/or elastic loading,^64^ as revealed from eq 1, which is the fundamental principle for monitoring the changes on the resonant frequency for a SAW device.^39^
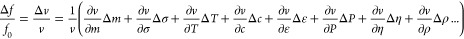
1where *f*_0_ is the central frequency of the SAW device, *v* is the phase velocity, Δ*m* is the change in mass, Δσ is the change in conductivity, Δ*T* is the change in temperature, Δ*c* is the change in mechanical constant, Δε is the change in the dielectric constant, Δ*P* is the change in pressure, Δη is the change in viscosity, and Δρ is the change in density.

**Figure 3:**
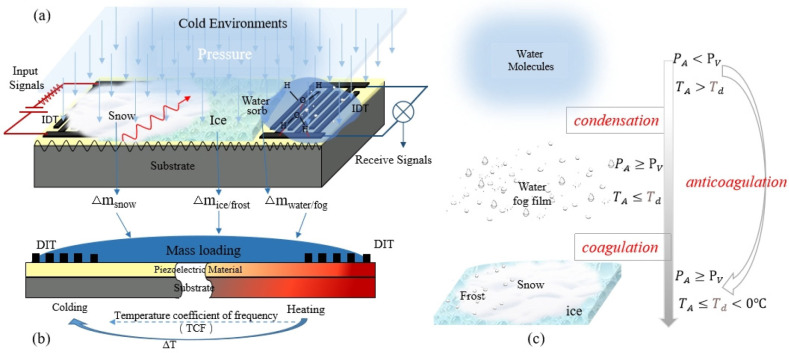
Effects of the environmental conditions on a SAW device. (a) Schematic illustration of water molecules, fog film, snow, and ice formed on the SAW surface. (b) Schematic representation of the mass loading and temperature effects arising from water molecules, fog film, snow, and ice formation on the SAW surface. (c) Schematic illustration of the process by which changes in the temperature and vapor pressure affect the water vapor to form fog and frost.

In a cold environment above the freezing temperature of the water, apart from the temperature change of Δ*T*, water molecules are usually absorbed on the surface of the SAW device, especially at high humidity, which mainly causes the mass loading effect,^46,47^ as shown in Figure 3a,b. Meanwhile, there are also other effects, such as changes in the impedance, conductivity, capacitance, etc., alongside the condensation or mass loading effect.^48,49^ With a further decrease of the temperature (e.g., temperature *T*_A_ drops below the dew-point temperature *T*_d_) and accessibility of saturation water vapor, the water vapor will be condensed into a water droplet or a layer of fog, causing a significant mass loading effect. As the temperature continues to drop (e.g., *T*_A_ ≤ *T*_d_ < 0 °C), the saturation water vapor or fog condensed from the saturation water vapor are then changed into ice/frost on the surface of the SAW device, as shown in Figure 3c. The formed ice could be in a rime type or glaze ice, which has dramatically different densities or other physical and functional properties. Because their thicknesses continuously increase with duration or a decrease of the temperature, the effects of mass loading will be enhanced. Figure 3 illustrates all of these changes, which result in different changes of the resonant frequencies for the SAW device.

Considering the complex influences of various parameters in a cold environment on the SAW device, in the following sections, we will first analyze each single factor and then discuss the influences of the combined or integrated factors using multivariate logistic regression analysis.

### 2.1 Temperature Influences

In a cold and dry environment, if only the temperature has been changed, the temperature coefficient of frequency (TCF) is often applied to show the rate of frequency changes versus temperature relative to the original resonant frequency of the SAW device. TCF is defined using eq 2:^42^
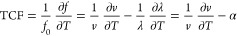
2where *T*, *f*, *v*, λ, and α are the temperature, frequency, phase velocity, designed wavelength, and temperature expansion coefficient of each material of the multilayer structure. The change of frequency (Δ*f*_*T*_) due to changes of temperature can be simply calculated using eq 3, which is a linear function of temperature.
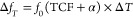
3

### 2.2 Mass Loading Effect

A mass loading on the SAW device’s surface arising from perturbations, such as the formation of a surface layer or adsorption of foreign objects, will change the wave propagation velocity, which is fundamentally related to the change in the wave energy density. A fundamental relationship between the wave velocity and energy density for the SAW device excited at a given frequency (or the fractional change in the wave velocity) is equal to the negative fractional change in the wave energy density.^50^ For example, when a layer of material is deposited onto a substrate that is vibrating synchronously with the waves, the changes of the average kinetic energy per area of the wave, Δ*U*_K_, can be described as
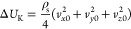
4
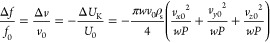
5where *v*_*x*0_, *v*_*y*0_, and *v*_*z*0_ are the SAW particle velocities at the surface, *f*_0_ is the central frequency, *v*_0_ and *U*_0_ denote the unperturbed wave propagation velocity and energy density, *w* is the angular frequency *w* = 2π*f*_0_, ρ_s_ is the density of the perturbation film, and *P* is the power density of the SAW device.

Considering the principle of wave propagation in a medium without external energy loss, the peak strain energy density will be equal to the peak kinetic energy density. Auld and Wohltjen simplified eqs 4 and 5 and derived the relationship between the resonant frequency of a SAW oscillator and the coating’s perturbation properties, which is described by the Wohltjen equation (9):^51,52^
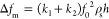
6where *f*_0_ is the central frequency, *k*_1_ and *k*_2_ are material constants of the SAW substrate (listed in Table 2), and *h* and ρ_s_ are the thickness and density of the coating perturbation film, respectively. The surface mass due to the adsorption of molecules or ice is related to *h*ρ_s_, i.e., Δ*m* = *h*ρ_s_*A*, in which *A* is the SAW device’s area. Therefore, an expression for the changes in the SAW frequency arising from the adsorption of water molecules or the formation of fog condensation can be derived.
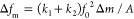
7

**Table 2:** Material Constants for Different SAW Substrates (Derived from Reference (51))

substrate	SAW velocity *v*_0_ (m/s)	*k*_1_ (m^2^·s/kg)	*k*_2_ (m^2^·s/kg)
Y cut X propagating quartz	3159.3	–9.33 × 10^–8^	–4.16 × 10^–8^
Y cut Z propagating LiNbO_3_	3487.7	–3.77 × 10^–8^	–1.73 × 10^–8^
Z cut X or Y propagating ZnO	2639.4	–5.47 × 10^–8^	–2.06 × 10^–8^
Z cut X or Y propagating Si	4921.2	–9.53 × 10^–8^	–6.33 × 10^–8^

Table 2 lists the material constants for a few selected SAW substrates,^51^ which can be used in the analysis based on eq 7.

**Figure 4:**
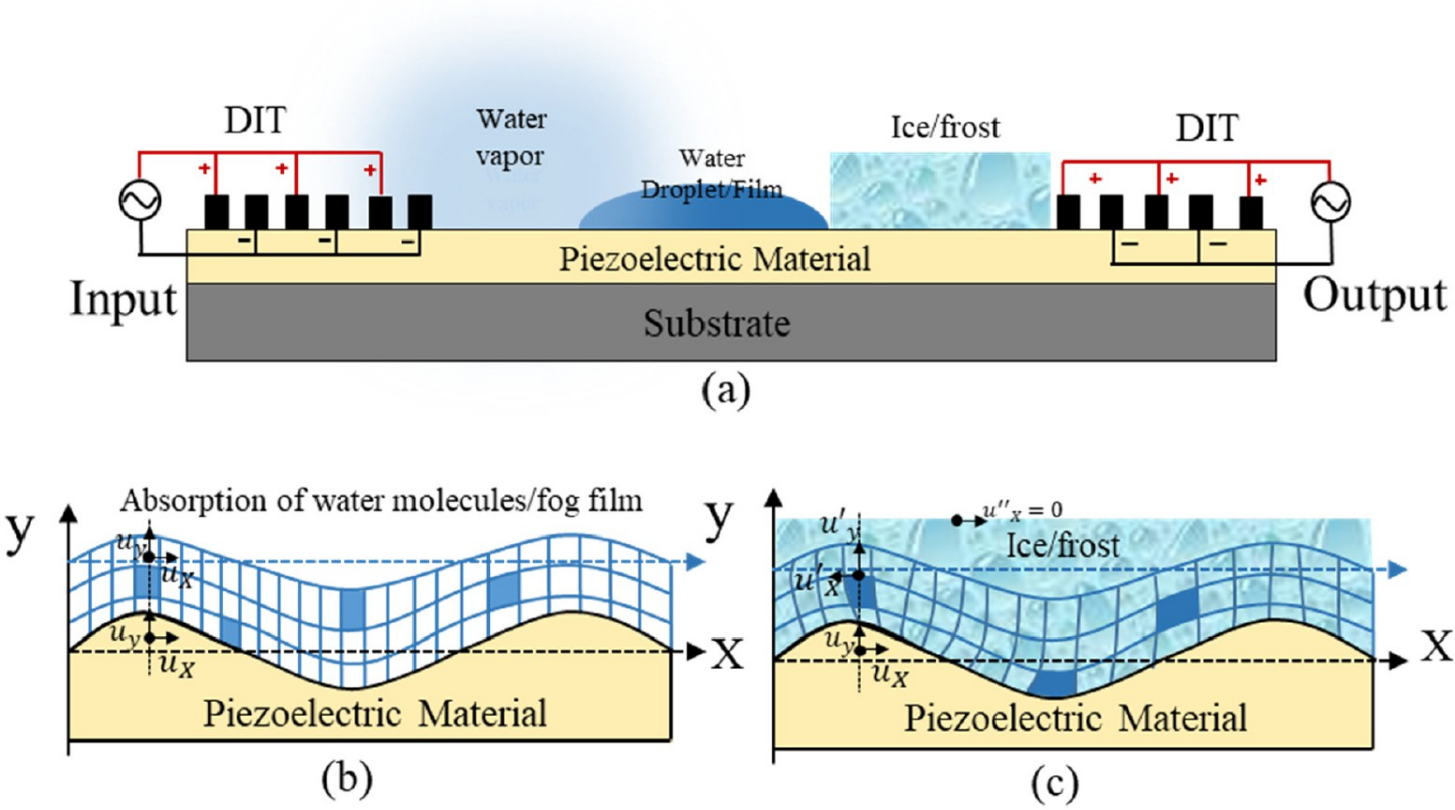
Perturbations on the surface of the SAW device and deformation generated by a SAW. (a) Perturbations for the SAW device in cold environments. (b) Schematic of water molecules/fog film moving synchronously with the wave. (c) Schematic of the thick and rigid ice layer film lagging behind the driven substrate/film interface.

However, it should be addressed that, in a cold environment, the mass loading effects arising from the adsorption of water molecules (or a fog layer formation) and the ice frozen on the surface of the SAW device are dramatically different, as illustrated in Figure 4a. For the former case, the adsorbed water molecules/fog layer is assumed to be sufficiently thin, as shown in Figure 4b, and the frequency effect can be estimated using eq 7. However, for a relatively thick and rigid ice layer, which is bonded well to the substrate, as shown in Figure 4c, large strains are generated across the thickness of the film. This results in nonuniform displacements across the ice film, which can be characterized by a function term related to the bulk and shear moduli (*K* and *G*) of the ice, *f*(*K*,*G*). Therefore, the changes in the SAW frequency arising from the ice layer perturbation can be calculated using eq 8:
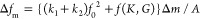
8where *f*(*K*,*G*) is a function of *K* and *G* of the ice. It should be emphasized that the bulk and shear moduli of ice changes with the density of ice and ambient temperature.^53^

### 2.3 Changes of the Electrical Properties

**Figure 5:**
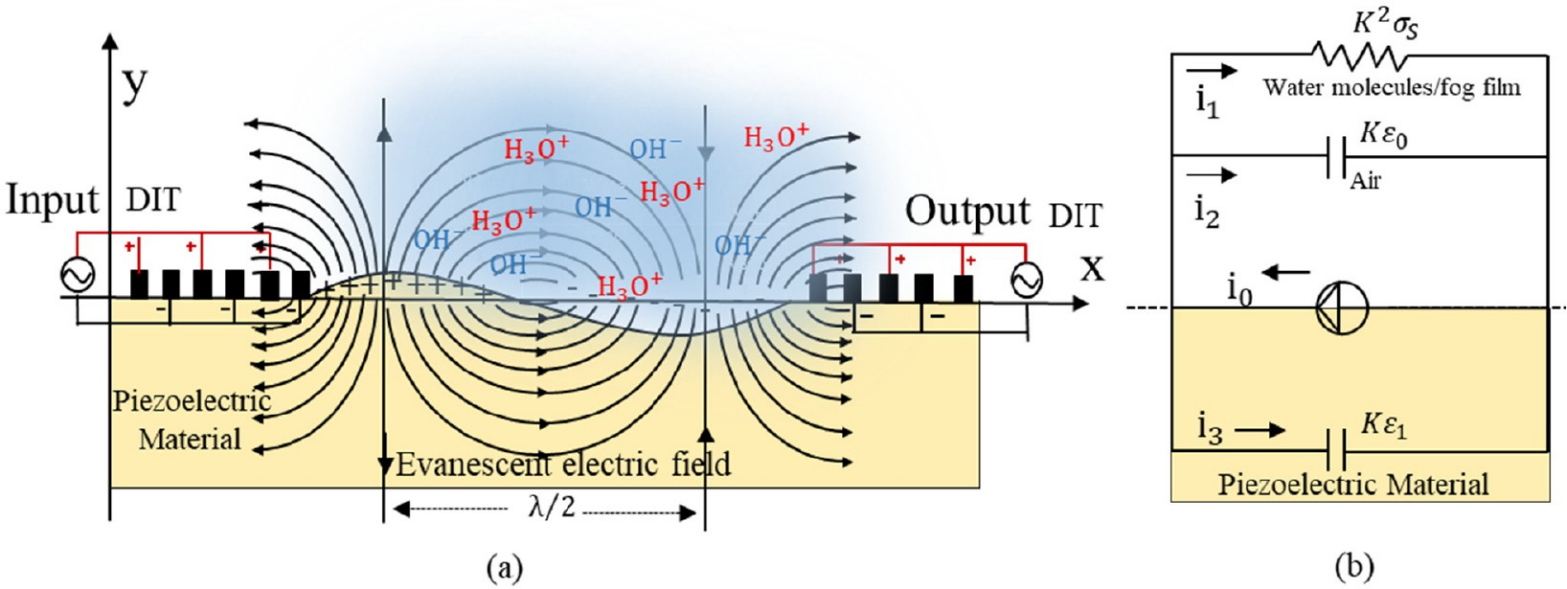
SAW-induced electrical property changes. (a) Evanescent electric field generated by a SAW propagating along the surface of a piezoelectric crystal. (b) Equivalent-circuit model to describe the acoustoelectric interaction between a SAW and water molecules/fog layer.

When an acoustic wave propagates in the piezoelectric material, it generates a layer of bound charges at the surface that arises from the generated strains. This induces an electrical polarization in the surface-normal direction of the crystal, accompanied by the propagating mechanical waves. Therefore, surface charges are sinusoidally varied with the charge density accompanying the mechanical waves, as shown in Figure 5a. The effect of wave/carrier coupling on the SAW propagation can be determined from a model that accounts for wave-generated conduction currents in the material and displacement currents in the adjacent dielectric media.^54^

The electrical loading effect is mainly caused by the absorption of water molecules on the surface of the SAW device, and it often happens in an environment where the atmospheric water vapor does not reach the saturation and dew-point temperatures. In this case, the atmospheric water vapor cannot be easily condensed into a droplet of water or an ice crystal^55,56^ on the surface of the SAW device. There are transfer phenomena of protons among water molecules that produces H_3_O ^+^ and OH^–^ under the applied electrical fields, as shown in formula (9):^57^
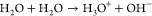
9

The hydrogen ions (protons) are transferred from one water molecule to another, thereby leading to changes of the electrical resistances.^49^ At the same time, the potential barriers and the active energy for conduction of the sensing materials are decreased by the adsorbed water, and then the amount of conducting electrons is increased.^57^ The resulting variation of the conductivity of the surface layer will change the velocity of the SAW device, thus leading to the frequency shift of the SAW sensor. The relationship between the frequency shift (Δ*f*_σ_) and surface conductivity (σ_s_) of the material can be described using eq 10:^58^
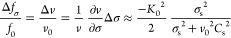
10where *K*_0_^2^ is the electromechanical coupling coefficient, *C*_s_ = ε_0_ + ε_s_, where ε_0_ and ε_s_ are the dielectric constants of air and the substrate, and σ_s_ = *h*ρ, where ρ is the resistivity and *h* is the thickness of the material. In the literature, there are reports that the electrical loading effect was caused by the adsorption of water molecules for nonspecific coatings such as Al_2_O_3_ nanotubes^59^ and nanocrystalline barium titanate,^60^ which influenced the phase and insertion loss of the SAW sensors.^50^

### 2.4 Cold Environmental Effects

In a cold environment, the phase transition of water vapor is related to the temperature and vapor pressure. When the saturated air is cooled, the moisture is released in the form of fog.^55^ When the saturated air comes in contact with a cold surface whose temperature is less than the dew-point temperature (i.e., above 0 °C, at which the air will be cooled to reach the saturation state at a constant-pressure condition) or the freezing temperature of water (0 °C or 273 K), the water vapor can be directly changed from a gaseous state to a solid state. It is well-known that the relationship between the temperature and saturation water vapor pressure is described by the following equation:^61^
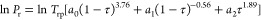
11where
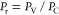
12
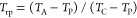
13
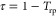
14
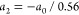
15where *P*_r_ is the reduced vapor pressure, *P*_V_ (Pa) is the saturated pressure at temperature *T*_A_ (°C), *T*_A_ is the absolute temperature, *P*_C_ (Pa) is the critical saturated pressure, *T*_rp_ is the reduced temperature, *T*_C_ is the critical temperature, *T*_P_ is the vapor temperature corresponding to a pressure of zero, and *a*_0_ and *a*_1_ are the parameters that are determined from the experimental data.^62^ For water, *T*_C_ = 647.14 K, *P*_C_ = 21898.54 kPa, *T*_P_ = 52.6 K, *a*_0_ = −3.506674, and *a*_1_ = 0.456054.

The conditions of a cold environment, such as water vapor, fog, and frost or ice, can be modulated by the ambient temperature and vapor pressure, whereas the vapor pressure is linked to the RH. RH is the ratio of the actual water vapor pressure to the saturation water vapor pressure at the prevailing temperature, calculated using the following equation:^63^
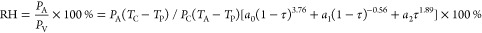
16where *P*_A_ (Pa) denotes the actual water vapor pressure value that is used to characterize the atmospheric water vapor content.

For the resonant frequency shifts of the SAW device, Δ*m*/*A* can be related to the RH value using the water absorption constant β, which is the mass of the adsorbed water molecules per unit RH and per unit area, typically expressed in units of kg/m^2^.
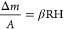
17

Combining eqs 7, 10, 17, and 3 with eq 1 yields the resonant frequency shifts of the SAW device due to the temperature change and formation of the water molecules/fog film:
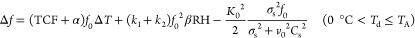
18where Δ*T* is the change of the temperature and *T*_d_ is the dew-point temperature. Combining eqs 8 and 3 with eq 1 yields an expression for the changes in the frequency for the SAW device arising from the temperature change and coverage of ice or frost:
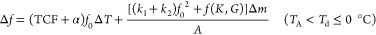
19

## 3 Experiments and Method

### 3.1 Preparation of SAW Devices

In this study, we selected ZnO thin-film SAW devices as an example for demonstrations for ice monitoring. The SAW devices were fabricated by depositing a piezoelectric ZnO film (∼3.5 μm thick) on aluminum plates (1.5 mm thick) and silicon and glass wafer substrates (both 500 μm thick) using radio-frequency magnetron reactive sputtering. During the deposition process, a zinc target with 99.99% purity was sputtered with a direct-current target power of 420 W, an Ar/O_2_ flow ratio of 10/13 sccm, and a gas pressure of 6 × 10^–4^ mbar. The distance between the target and substrate was 100 mm, and the substrate was rotated during deposition to improve the uniformity of the deposited film without intentional substrate heating.

The interdigitated transducers (IDTs) of the SAW devices were fabricated by using a conventional photolithography and lift-off process, with a bilayer of 20 nm Cr and 100 nm Au. SAW devices with different IDT wavelengths (e.g., 64, 100, 200, and 300 μm) were prepared and used in different experiments (as listed in Table 3). One example of the fabricated ZnO/Al SAW devices can be found in Figure S1. A network analyzer (Agilent E5061B) was used to characterize the reflection (S11) spectra of the SAW devices, with one example shown in Figure S2.

**Table 3:** SAW Devices Used for All of the Experiments

device number	thin film/substrate	central frequency (MHz)	wavelength (μm)
1	ZnO/Al	9.64	300
2	ZnO/Al	14.27	200
3	ZnO/Al	27.26	100
4	ZnO/Al	40.10	64
5	ZnO/glass	14.97	200
6	ZnO/glass	23.09	100

### 3.2 Characterization of SAW Sensors in Cold Environments

First the frequency changes of SAW devices 1–4 were recorded by cooling them from +25 to −20 °C in an open environment without controlling the humidity, the method of which was previously introduced in ref (64). In the test, a semiconductor cooler was used to control the substrate temperature, and a K-type thermocouple (CO2-K, OMEGA Engineering) was fixed on top of the SAW device for temperature measurements (Figure S3).

**Figure 6:**
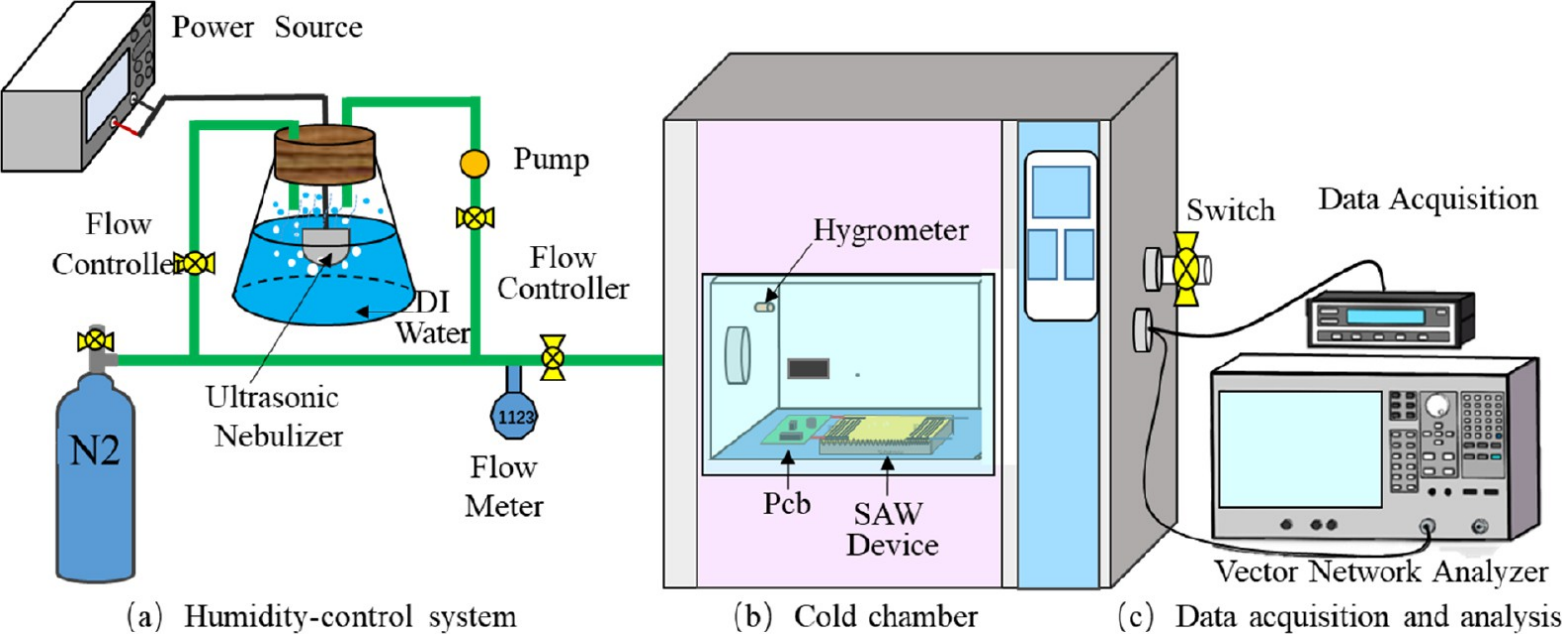
Schematic illustration of the cooling/humidity control system: (a) system for humidity control; (b) cold chamber with adjustable temperatures; (c) data acquisition and analysis system.

To monitor the frequency shifts of ZnO thin-film SAW devices in a controlled laboratory environment, a cold chamber (Advanced test systems, China, LH1000-LN2) with adjustable temperatures via the current input and precise humidity control was used (Figure 6). The ZnO thin-film SAW device was directly connected to the cold plate inside the cold chamber to ensure good contact. A thermocouple was also attached to the ZnO thin-film SAW device for temperature measurement, and a humidity sensor was placed inside the cold chamber for the RH measurement during operation. During all of the tests, the SAW device was placed in the cold chamber and the frequency responses of the ZnO thin-film SAW devices were recorded in real time.

To evaluate the temperature effects of the ZnO thin-film SAW devices (devices 1–4 were chosen) with a minimized humidity effect (e.g., the RH value is maintained at ∼0%), the cold chamber was cooled from temperature ∼80 ± 0.3 °C (a dry and hot environment) to a set low temperature of ∼−20 ± 0.1 °C (a dry and cold environment) and then gradually increased from −20 to +80 °C at a rate of 10 °C/min. In this test, both the frequency and temperature were recorded simultaneously.

To evaluate the humidity effects on the ZnO thin-film SAW devices, we conducted experiments about the humidity effects in the cold chamber by performing tests using device 2 at different RH values from 10% to 90% but at four different temperatures, e.g., −4 °C (a wet and cold environment) and 0, 4, and 10 °C (a wet and warm environment), respectively. The RH values were controlled by flowing the chamber with different ratios of humid air and nitrogen carrier gas. Five readings of the frequency values were in situ recorded after the SAW device was kept in a stable humidity value for ∼20 min, and the average values were taken.

To evaluate a combined effect of humidity and temperature on the ZnO thin-film SAW devices, we first examined the relationship between the temperature and RH values in the enclosed cold chamber to reduce any analysis errors of the independent factors (i.e., RH level and temperature) and then studied their combined influences on the frequency shifts of the SAW devices. In the test, the starting RH value was adjusted to 60% and 80%, and the starting temperature was adjusted to +60 °C (i.e., a warm and wet environment) and −10 °C (i.e., a cold and wet environment). Then the RH values were recorded with the temperature decreased at a rate of 3 °C/min. The dynamic frequency shifts (Δ*f*) of the SAW device as a function of both the temperature and RH were also performed. Both SAW devices 1 and 4 were kept in the environmental chamber with a humidity of ∼0% by cooling the temperature from +30 to −45 °C at a rate of 3 °C/min and then maintained at −45 °C for ∼1 min. The chamber was then opened, and the ambient air (which had a room temperature of 20 °C and a humidity of 65%) was introduced to the chamber for 1 min. The temperature of the controlled chamber was then naturally increased to 25 °C when the door was opened. During all of these procedures, the frequencies of the SAW devices were continuously recorded.

Finally, the frequency shifts (Δ*f*) of SAW devices with varied ice thicknesses and ice types (i.e., rime ice or glaze ice) were monitored. For the ice thickness tests, SAW device 1 was cooled along with the chamber at RH = 100% (i.e., by blowing with cold humid air) and different temperatures of −10, −20, −30, and −40 °C. The thickness of rime ice (formed from small droplets or fogs of water with a rough, opaque surface and brittle tissue)^38^ was estimated by weighing the mass of the device before and immediately after the icing process at 1 min intervals. To do different ice type tests, SAW devices 5 and 6 were cooled in the chamber with RH = 80% and the temperature was set at −10 °C, and then the glaze ice (formed from large droplets with smooth, transparent surfaces and dense tissues)^38^ was formed by freezing a deionized water droplet on the SAW devices’ surface within a poly(dimethylsiloxane) chamber (with internal and external sizes of 55 × 30 × 5 mm and 65 × 40 × 5 mm, respectively) and further frozen for 10 min to increase the adhesion process. In these tests, the frequency readings were recorded continuously (Table 4).

**Table 4:** Different Cold Testing Conditions in This Study

test number	manner of the experiment	devices
1	open environment without controlling the humidity	1–4
2	temperature effects; RH = ∼0%	1–4
3	stable humidity for ∼20 min	2
4	association between the temperature and RH in the enclosed cold chamber	
5	dynamic influence of both the temperature and RH	1 and 4
6	influence of ice thickness, with RH = 100% and temperatures of −4, −10, and −20 °C for 20 min	1, 5, and 6
7	influence of ice type	1, 5, and 6

## 4 Results and Discussion

### 4.1 Influence of a Single Factor

**Figure 7:**
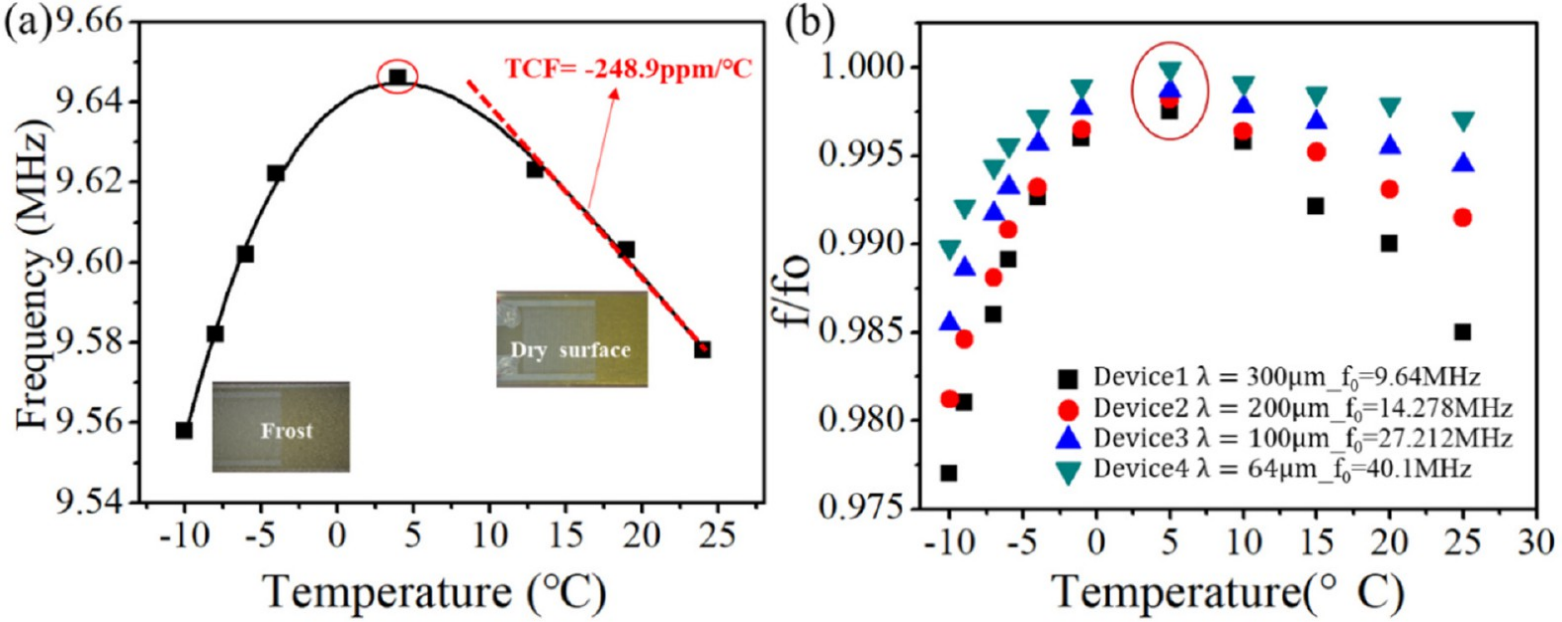
Variations of the resonant frequency of the SAW device as a function of the temperature in an open environment. (a) Plots of the resonant frequency and temperature for a SAW device with λ = 300 μm and *f*_0_ = 9.64 MHz at different temperatures in the open environment. (b) Comparison curves and plots of the resonant frequencies of SAW devices with different wavelengths changing with temperature (temperature interval where the phase transition of water vapor occurs).

Figure 7a shows variations of the resonant frequency of SAW device 1 at different temperatures obtained in an open environment. As shown in this figure, when the temperature is gradually decreased, the frequency of the SAW devices is increased; e.g., the resonant frequency of the SAW device increases with a decrease of the temperature. The calculated TCF value is at about −248.9 ppm/°C for this ZnO/Al SAW device, which shows a reading similar to that reported in ref^40^ for a similar SAW device. However, a turning point is observed (e.g., with a maximum value of the frequency marked with a circle in Figure 7a) at ∼2–5 °C. With a further decrease in the temperature, the frequency was decreased significantly. Similar patterns of frequency changes for the SAW devices during cooling were frequently reported in the literature, i.e., refs (64−66), whereas the explanations for this phenomenon have not been previously provided. We believe that this phenomenon is predominantly caused by the significant absorption of water molecules (or formations of condensed moisture) at a low temperature just above 0 °C, which dramatically decreases the frequency values.

Figure 7b shows the resonance frequency changes of SAW devices with different wavelengths as a function of the temperature, where all of the curves show that there is a turning point at which the phase transition of condensed water occurs, a few degrees just above 0 °C. As the temperature is further decreased, the resonant frequencies of SAW devices for all samples exhibit decreasing trend. Moreover, it was observed that the smaller the wavelength is (or the higher the frequency is), the faster the change rate of the frequency–temperature curves (Figure 7b), indicating that higher-frequency devices would be more sensitive to moisture/ice loading.

**Figure 8:**
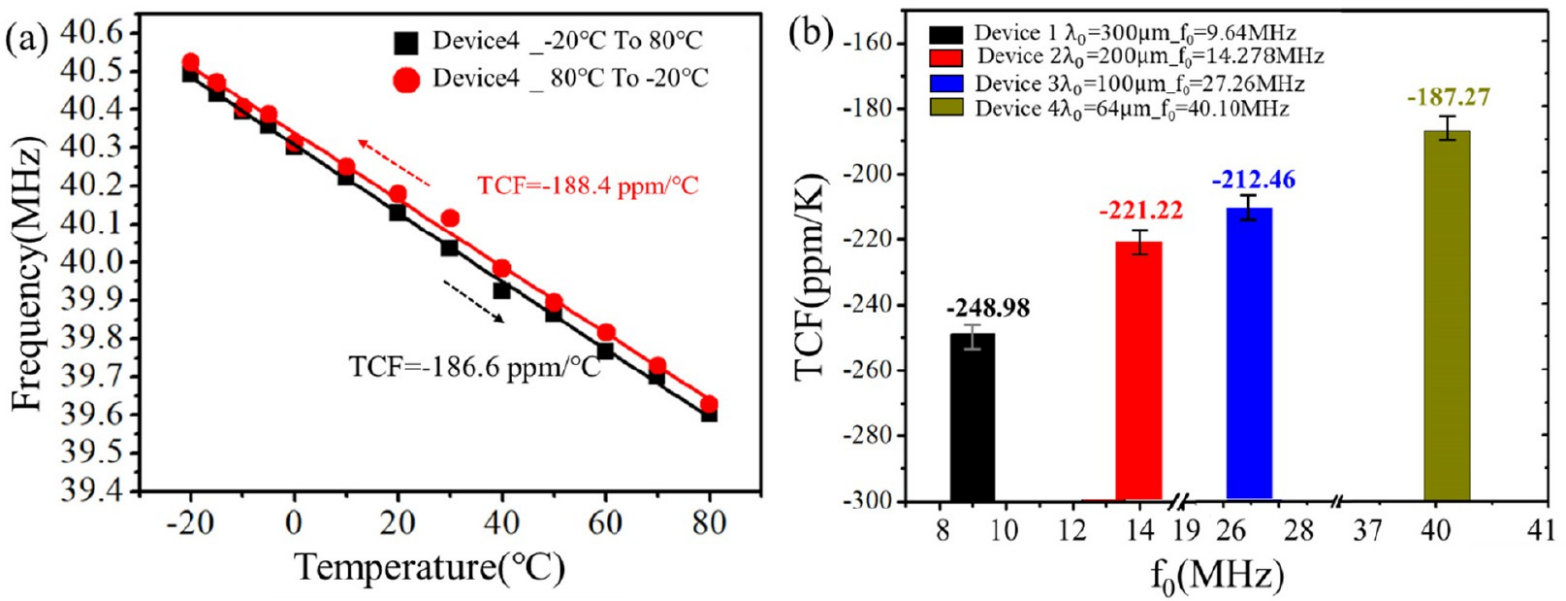
Variations of the resonant frequency of the SAW device as a function of the temperature in a nearly RH = 0% environment. (a) Plots of the resonant frequency and temperature for SAW device 4 in the drying experiment. (b) Statistical graphs of the TCF for the same structure (the same size of each layer constitutes the SAW device) and different designed wavelengths (different sizes of spacing of fingers) of the SAW devices.

To study the real principle for this phenomenon, the changes of the frequencies for SAW device 4 as a function of the temperature during the cooling and healing stages with a controlled RH value of nearly 0% (i.e., in a dry condition) were measured. The obtained frequency–temperature curve is shown in Figure 8, which shows an approximately linear trend. All other tested devices with different resonant frequencies under dried conditions show a linear trend under such dry testing conditions (Figure S4). The TCF values were calculated based on these linear lines, and the results are summarized in Figure 8b. The higher the resonant frequency (or the smaller the wavelength), the smaller the absolute values of the TCF values, which are similar to those reported in ref (42).

**Figure 9:**
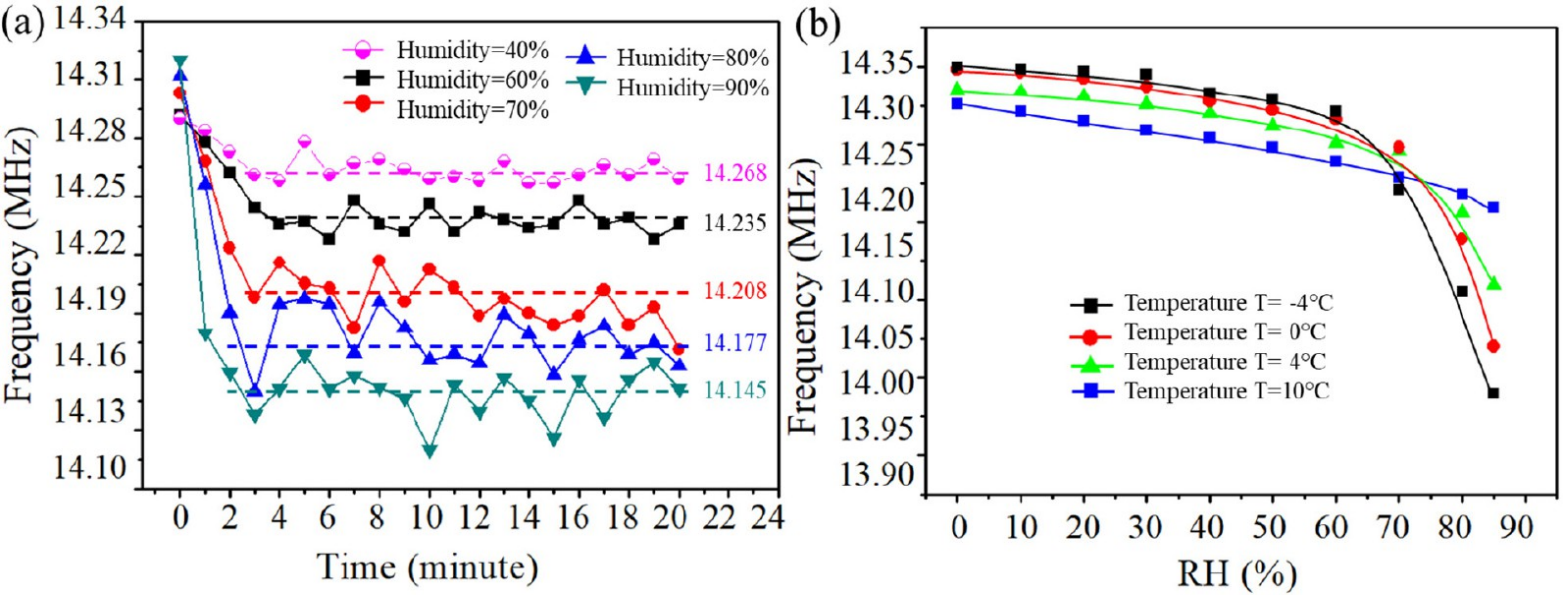
Plots of the temperature and humidity changes for the SAW device. (a) Plots of the resonant frequency and humidity for SAW device 4 with λ = 200 μm and *f*_0_ = 14.278 MHz, which was kept in an environmental chamber at relative RH values of 40%, 60%, 70%, 80%, and 90%, respectively, and a low temperature of 6. The process was realized by blowing humid air into the chamber to cause dynamic changes of the resonant frequencies (thus causing large variations of the data). (b) Plots of the resonant frequency and humidity for a SAW device with λ = 200 μm and *f*_0_ = 14.278 MHz at different temperatures of −4, 0, +4, and +10 °C, respectively.

Figure 9a shows the results of the resonant frequency changes of the SAW devices as a function of the RH values. Significant decreases of the frequencies were observed at the starting stage at a temperature of 6 °C, but the decrease rate of the frequency was found to reduce slowly with time, finally reaching a stable value. The SAW devices were further tested at temperatures of −4, 0, +4, and +10 °C, respectively. It was observed that, with an increase of the RH level, there is a more dramatic decrease of the frequency at the initial stage, which is attributed to the adsorption of more water molecules at the higher RH level during that period. From Figure 9a,b, we can conclude that both the temperature and RH level have significant influences on the frequency changes of the SAW devices.

### 4.2 Dynamic Changes of Both the Temperature and Humidity

**Figure 10:**
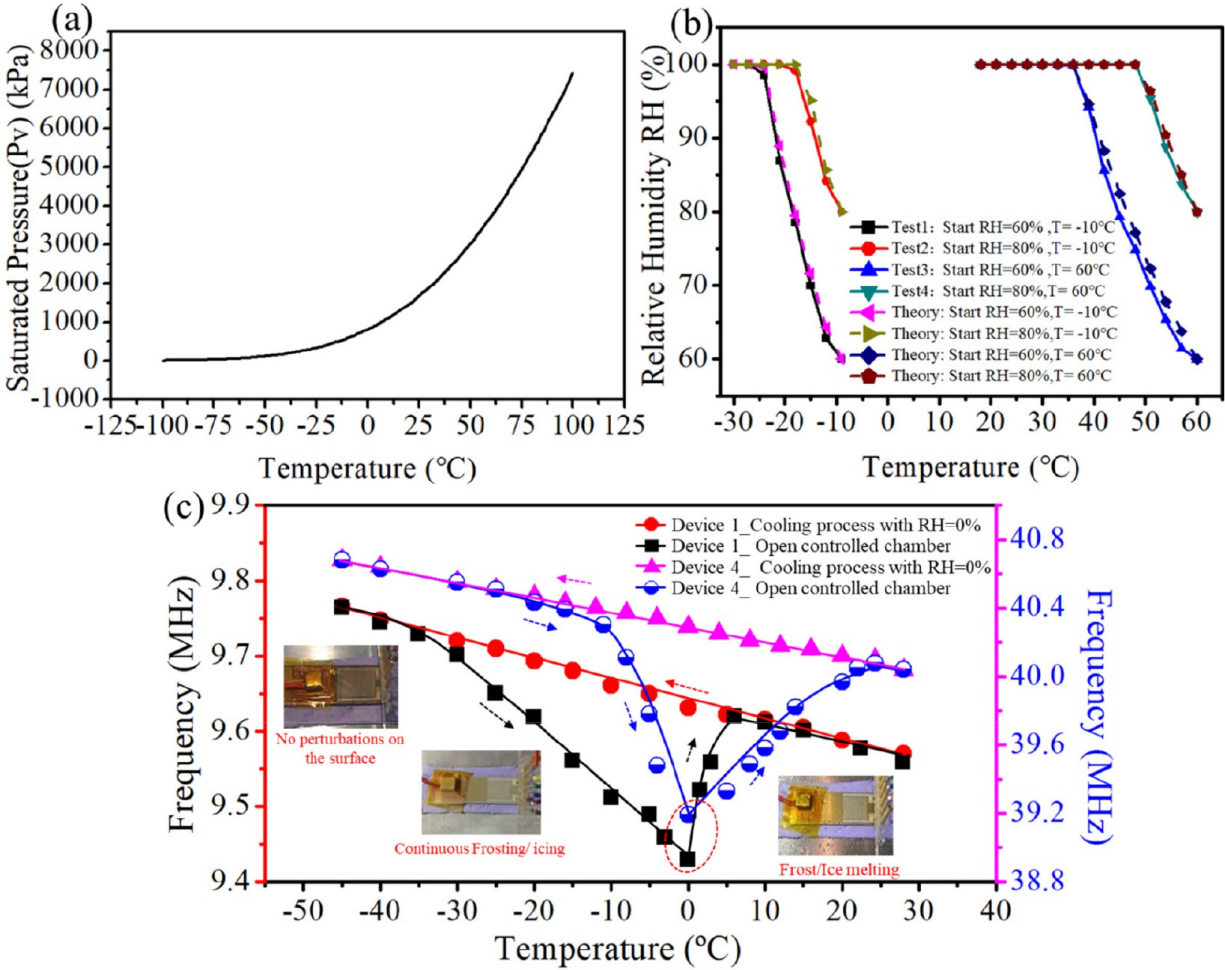
(a) Theoretical curve for the temperature and vapor pressure in the enclosed cold chamber according to eqs 11–15. (b) Variations of the RH with temperature at starting RH = 60% and 80% and different temperatures of *T* = −10 and 60 °C, respectively. The dashed lines are the fitting curves based on the theoretical calculation. The solid curves are the fitting curves based on the experimental data. (c) Plots of the resonant frequency and temperature for the SAW devices in drying and frosting experiments, where the red and black curves correspond to the left axis and the pink and blue curves correspond to the right coordinate values.

The experimental data thus far indicate that combined effects of the temperature and RH have strong influences on the frequency changes of the SAW devices. Figure 10a shows fitted curves using calculated values of the saturated water vapor pressures and temperatures based on eq 11, which indicates that the saturated pressure (*P*_V_) of the water vapor decreases as the temperature decreases.

Figure 10b shows that the RH values are changed with the temperature, in which the experimental results (solid curves) matched well with the theoretical results obtained using eq 16 (i.e., dashed lines). As shown in Figure 10b, in a cold environment (e.g., at an initial temperature of −10 °C), when the initial water vapor content (*P*_A_) is 60%, the decrease of the temperature from *T* = −10 to −22 °C (Δ*T* = 12 °C) results in a decrease of the saturated vapor pressure (*P*_V_), and then the RH (RH = *P*_A_/*P*_V_) is increased to full saturation (RH = 100%). As shown in Figure 10b, when the starting water vapor content (*P*_A_) is 60%, as the temperature is decreased from *T* = −10 to −22 °C (Δ*T* = 12 °C) in a cold environment, the RH value is increased to full saturation (RH = 100%). In order to achieve full saturation for an initial *P*_A_ of 80%, the temperature only needs to drop by 7 °C from *T* = −10 to −17 °C. However, in a warm environment (e.g., with an initial temperature of 60 °C), when the initial *P*_A_ is 80%, in order to achieve full saturation, the temperature needs to decrease from *T* = 60 to 47 °C, that is, requiring a temperature reduction of 13 °C. In the warm environment with an initial *P*_A_ of 60%, the temperature needs to be reduced by 22 °C (from *T* = 60 to 38 °C) for the RH to reach saturation (RH = 100%).

Figure 10c shows the results of resonant frequency changes for SAW devices 1 and 4 when they were placed in an environmental chamber where both the humidity and temperature were dynamically changed. In the process of cooling where the humidity was very low, the resonant frequencies of both SAW devices 1 and 4 were increased linearly with a decrease of the temperature due to pure thermal/temperature effects. As shown in Figure S6a, no apparent ice formation was observed on the surface of devices. However, during the heating stage, when ambient air (20 °C and RH of 65%) was suddenly introduced into the controlled chamber and the chamber temperature was quickly increased from −45 to 0 °C, frost began to form on the SAW devices’ surfaces (as shown in Figure S5b). The thickness of this frost layer was gradually increased as the freezing temperature was continuously increased in the cold environment (i.e., below 0 °C). With frost formed on surfaces and a continuous increase of the frost thickness, the frequency of the SAW devices was found to decrease significantly with increases in temperature (e.g., the blue and black curves shown in Figure 10c). However, as the temperature was increased above 0 °C, there was a sharp change of the curves for the frequency of the SAW device, which is mainly because the frost began to melt and the mass loading effect was significantly reduced with quick evaporation of the water (as shown in Figure S5c). This dramatically changes for the frequency curves of the SAW devices and could be explored to monitor evolutions between frost/ice and moisture formation. After the surfaces were gradually dried, the resonant frequencies of both devices 1 and 4 were decreased linearly with an increase of the temperature.

### 4.3 Ice Formation on the SAW Device

**Figure 11:**
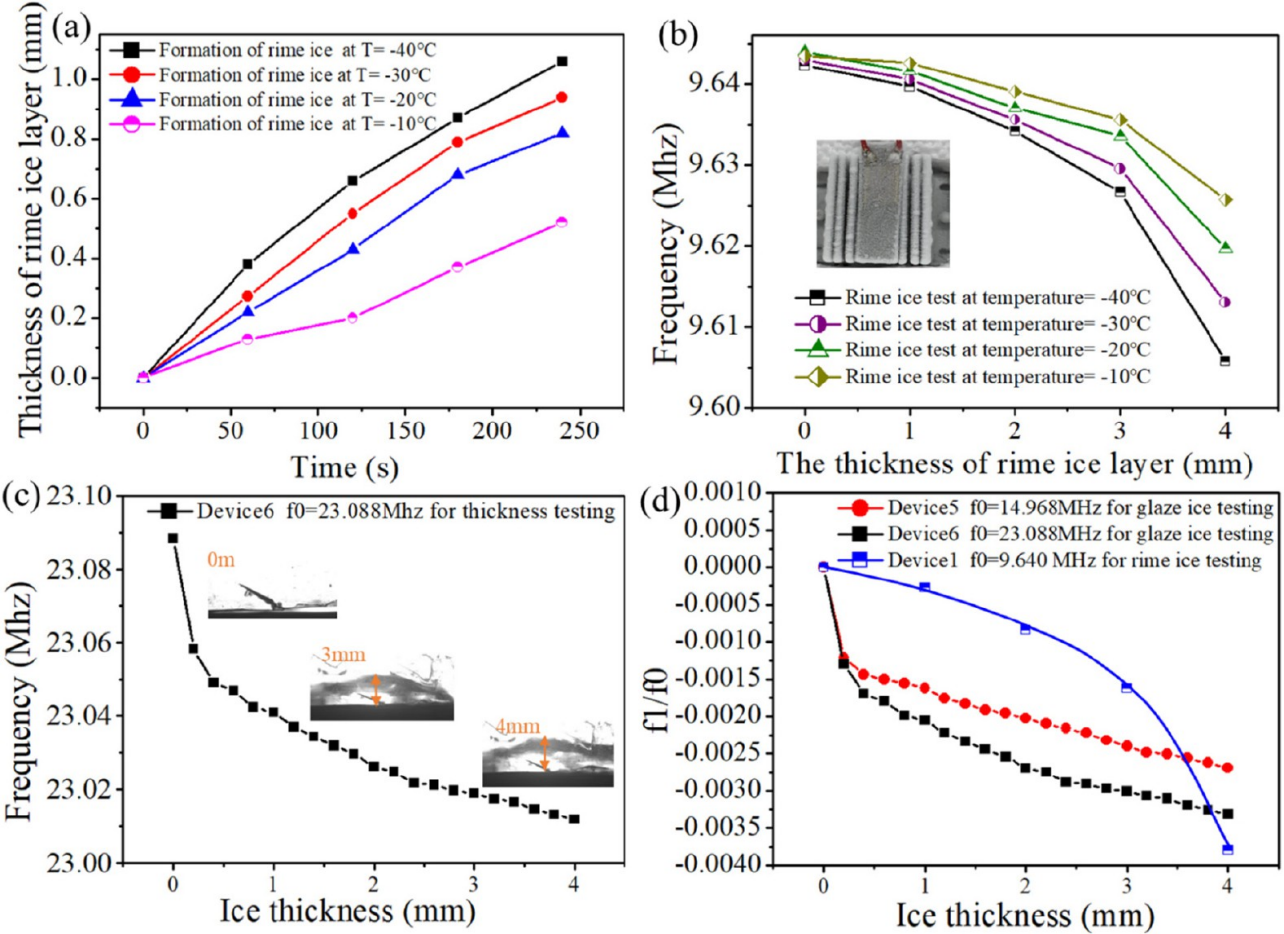
Influences of the ice layer thickness and ice types on the SAW devices. (a) Curves of the rime ice thickness and freezing time at different temperatures in ice thickness tests. (b) Plots of the frequency and rime ice thickness at different temperatures. (c) Curves of the frequency and glaze ice thickness at a temperature of −10 °C. (d) Comparison curves of the influence degrees for different ice types and ice thicknesses on the SAW frequency.

Parts a and b of Figure 11 show that, with the continuous inflow of saturated water vapor, the lower the set temperature in the chamber, the faster the decrease for the frequency of the SAW device, which is mainly due to the faster generation speed of the rime ice on the surface of the SAW device. Figure 11c shows that the frequency of the SAW device decreases with an increase in the glaze ice thickness. As shown by the black and red lines in Figure 11d, when the thickness of the rime ice increases, the frequency decreases slowly at the beginning stage, and then this decrease rate becomes significant later. However, the trend is quite different for the glaze ice, as shown by the blue line in Figure 11d. When the thickness of the glaze ice is increased, the frequency initially decreases sharply but then becomes much slower later.

## 5 Discussion

**Figure 12:**
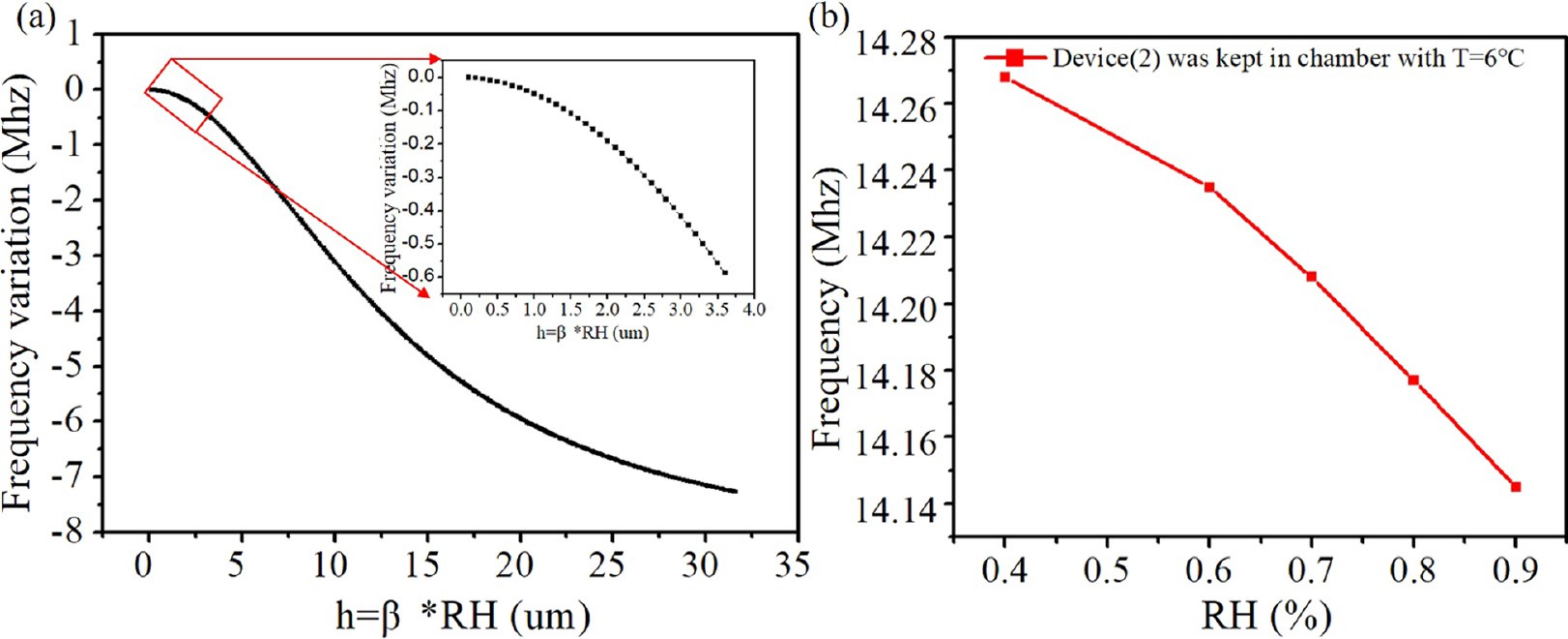
Experimental data and theoretical fitting curves for the influence of the single factor of humidity on the frequency of the SAW device. (a) Theoretical curve fitted according to eq 20. The black dashed line is a local amplification of the theoretical fitting curve. (b) Red curve showing the fitting to the experimental data of SAW device 2 in a warm and humid environment where the temperature is 6 °C.

From the results of the experiments, the effect of temperature on the frequency of the SAW sensor is linear at a dry condition, and the rate of change can be calculated based on the TCF. The effect of humidity on the frequency values of the SAW sensor is nonlinear, and the frequency changes become more significant with an increase of the humidity, as shown by the red line in Figure 12. According to the theoretical model, i.e., eq 16, at this time, i.e., Δ*T* = 0 (the temperature was maintained constant at 6 °C), the surface conductivity (σ_s_) of the adsorption of water molecules can be written as σ_s_ = *h*ρ = (βRH)ρ. The influence of humidity on the frequency of the SAW device can be obtained using eq 20:
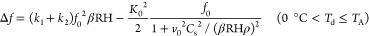
20where *k*_1_ = −5.47 × 10^–8^ m^2^·s/kg, *k*_2_ = −2.06 × 10^–8^ m^2^·s/kg, *f*_0_ = 14.25 MHz, *K*_0_^2^ = 1.2%, ρ = 1900 Ω/m, *v*_0_ = 2649 m/s, and *C*_s_ = ε_0_ + ε_s_ = 8.5 + 1 = 9.5 Pf/cm.^50,66^ Based on the above formula (i.e., eq 20) and the related theoretical parameters, the variation trend of the frequency of SAW and RH can be obtained by using the variable values.

Figure 12 shows the experimental data and theoretical fitting curves for the influences of the single factor of humidity on the frequency of the SAW device. The thick black line in Figure 12a shows the theoretical curves obtained using eq 20. By qualitative analysis, the absolute changes of RH and Δ*f* present the nonlinear and monotonic decreases. The results of the theoretical model (i.e., using eq 20 agrees well with the experimental results of the influence of the single factor of humidity on the frequency of the SAW device, as shown in Figure 12b). Therefore, in a high-humidity environment, the effect of the humidity on the SAW sensors should be analyzed by combining the effects of mass loading and surface charging.

From the results of the dynamic experiments (Figure 10), temperature changes will result in changes in the RH levels by affecting the saturated water vapor pressure. Because the water vapor content (*P*_A_) is fixed, the saturated vapor pressure (*P*_V_) will be decreased with a decrease of the temperature. However, because the water vapor content remains unchanged, the RH will be increased with a decrease of the temperature. When the temperature drops below the dew point (*T*_A_ ≤ *T*) and the supersaturated condition of water vapor (RH ≥ 100%) is reached, the water vapor is condensed quickly onto the surfaces of SAW devices. The mass loading effect becomes more pronounced for changing the frequencies of the SAW devices. When the temperature is continuously decreased (*T*_A_ ≤ *T*), more water vapor in the air is condensed and frozen as frost or ice, and thus the frequency will be decreased even faster.

Figure 10c shows the influence of phase changes for water vapor on the frequency shifts of SAW devices in a cold environment. When the surface of the SAW device is covered with frost/ice, as the temperature is increased from −45 to +20 °C, the frost/ice is melted and the water mist is evaporated. Therefore, there are significant frequency changes of the SAW device during dynamic changes of ethe nvironment from a dry and cold environment to a wet and cold environment and then to a hot and wet environment. The theoretical models, e.g., eqs 8, 18, and 19, are used for analysis of the influences of the phase change for water vapor and the formation of frost/ice on the frequency shifts of SAW devices. Because the bulk and shear moduli (i.e., *K* and *G*) of ice cannot be precisely obtained, the change trend cannot be analyzed quantitatively. The frequency shifts of SAW devices in the wet and cold environments due to the coverage of frost/ice are due to the results of the joint effects of temperature (Δ*T*) and RH (ΔRH). As the external warm and wet air is slowly introduced in the cold chamber, the temperature is gradually increased (*T*_A_ < 0 °C). The changes of Δ*T* cause changes of the device’s frequency. Simultaneously, due to the mass loading effect, the mass change Δ*m* is also continuously increased. The absolute value of frequency Δ*f* increases with an increase of Δ*T* and also with the mass loading Δ*m*; therefore, the frequency of the SAW device shows a substantially decreasing trend. As the temperature is continuously increased (*T*_A_ ≥ 0 °C), frost/ice begins to melt and evaporate, and the frequency of the SAW device is gradually increased due to the significantly reduced mass loading effect. Once all water or vapor on the SAW device is removed, a linear trend will be seen due to the dominant temperature effect (Δ*T*). Qualitative analysis of the theoretical model is consistent with the curve trend fitted with the experimental data, as shown in Figure 10c.

From the results of the ice thickness and ice type experiments, the frequency changes of SAW devices are sensitive to the ice types and ice thickness. The frequency decreases nonlinearly with increasing ice thickness (i.e., for both the rime ice and glaze ice). According to the theoretical model (i.e., eq 16), because the linear changes in the frequency caused by the mass loading (Δ*m*) due to an increase in the ice thickness are superimposed with the nonlinear changes in the frequency caused by the changes of the *f*(*K*,*G*) term due to the bulk and shear moduli (*K* and *G*) of the ice, the final frequency changes of the SAW devices are decreased nonlinearly with the ice thickness. Because the density of the glaze ice (exceeding 900 kg/m^3^) is much higher than that of the rime ice (200–600 kg/m^3^),^67,68^ the mass loading effect due to the glaze ice at the same thickness is much more significant than that for the rime ice. The rime ice is in loose contact with the surface of the SAW device, and the ice particles cannot effectively vibrate along with the SAW device. A positive frequency shift effect for the SAW device during the initial stage of frost and ice formation will weaken the mass loading effect for SAW devices. Therefore, the frequency of SAW devices decreases slowly at the beginning stage, and then this decrease rate becomes much larger due to mass accumulation and compaction of the ice. However, for the glaze ice, the bonding between the glaze ice and the substrate is stronger, so the interface can be considered to be a rigid contact. Thus, the bulk and shear moduli (*K* and *G*) of the ice become more important, which can cause large strains and nonuniform displacement across the glaze ice/substrate to consume the energy of SAW transmission and reduce the propagation speed.^69^ Therefore, the rigid contact and mass loading effect work together and result in the frequency of the SAW device decreasing sharply at the beginning stage and then much less sharply later for glaze ice.

## 6 Conclusions

In this study, we systematically studied the resonant frequency changes based on the adsorption of water molecules, moisture condensation, and frost/ice formation by considering the temperature, electrical loading, and mass loading effects for SAW devices in cold environments. Experiments were then carried out to study the influences of the temperature and/or humidity as well as their hybrid effects on the evolution of the frequencies of SAW devices. The mechanisms and influential processes of the phase transition caused by water vapor in dynamic environments of dry and cold, dry and hot, warm and wet, and cold and wet on the frequency of SAW devices were systematically investigated. The theoretical models that qualitatively predict the production of phenomena such as moisture condensation and formation of fog, frost, and ice in a cold environment are obtained. Both the experimental results and theoretical analysis demonstrated that the absorption of water molecules is the main reason for the nonlinear changes of the SAW frequency with the temperature at room temperature. As the temperature drops below the dew point (*T*_A_ ≤ *T*), water molecules precipitate to form fogwater, which intensifies the decreasing trend of the SAW frequency. Compared with those for the rime ice, the effect of the bulk and shear moduli (*K* and *G*) of the glaze ice on the shift of the SAW frequency is more significant at the initial stage of formation. More importantly, the frequency changes of SAW devices are sensitive to the ice types and ice thicknesses, which can be used to monitor different ice types and ice thicknesses. Therefore, all of the theoretical and experimental results in this study lay a solid foundation for developing high-precision SAW icing detection sensors.

## Supporting Information Available

Schematic diagram of the SAW surface device, measurement system of the reflection spectrum (S11) of the SAW device, temperature control on semiconductor refrigerators for SAW devices, and plots of the frequency and temperature for the SAW device (PDF)

## Abbreviations

*f*_0_: central frequency of the SAW device
λ: SAW wavelength
*v*: propagation velocity of the SAW devices
Δ*m*: change in mass
Δσ: change in conductivity
Δ*T*: change in temperature
Δ*c*: change in mechanical constant
Δε: change in the dielectric constant
Δη: change in viscosity
TCF: temperature coefficient of frequency
Δ*U*_K_: change in the average kinetic energy of the SAW device per area
*U*_0_: energy density
*w*: angular frequency
*ρ*_s_: density of the perturbation film
*P*: power density of the SAW device (power/area)
*k*_1_: material constant of the SAW substrate
*k*_2_: material constant of the SAW substrate
*h*: thickness of the coating perturbation film
*A*: surface area of the SAW device
*K*: bulk moduli of ice
*G*: shear moduli of ice
*K*_0_: electromechanical coupling coefficient
ε_0_: dielectric constant of air
ε_s_: dielectric constant of the substrate
σ_*s*_: surface conductivity of the perturbation material
ρ: resistivity of the perturbation material
*P*_A_: actual water vapor pressure
*P*_C_: pressure at the critical point
*P*_r_: reduced pressure
*P*_V_: saturated vapor pressure
*T*: absolute temperature
*T*_A_: absolute temperature
*T*_C_: temperature at the critical point
*T*_d_: dew-point temperature
*T*_r_: reduced temperature
*T*_p_: vapor temperature corresponding to a pressure of zero
*T*_rp_: reduced temperature of the new vapor equation
β: mass of the adsorbed water molecules per unit RH and per unit area
