# Supplementary material for: Fundamentals of Monitoring Condensation and Frost/Ice Formation in Cold Environments Using Thin-Film Surface-Acoustic-Wave Technology
Source: ACS Appl Mater Interfaces. 2023 Jul 11;15(29):35648–63. doi: 10.1021/acsami.3c04854 (PMC10375437; doi:10.1021/acsami.3c04854)
Supplement: Supplementary file 1 — am3c04854_si_001.pdf [file am3c04854_si_001.pdf]

Supplementary Information

Fundamentals of monitoring condensation and frost/ice formation in cold environments using thin film surface acoustic wave technology

Xingchang Zeng[1,2], Huiling Ong[3], Luke Haworth[3], Yuchao Lu[1], Deyu Yang[4], Mohammad Rahmati,[3] Qiang Wu,[3] Hamdi Torun,[3] James Martin,[3] Xianghui Hou[4], Xianglian Lv [1], Weizheng Yuan [1], Yang He,[1], Yongqing Fu*,[3]*

[1] Key Laboratory of Micro/Nano Systems for Aerospace, Ministry of Education and Shaanxi Key Laboratory of Micro and Nano Electromechanical Systems, School of Mechanical Engineering, Northwestern Polytechnical University, Xi'an 710072, P. R. China

[2] Xi'an institute of applied optics, Xi'an 710072, P.R.China

[3] Faculty of Engineering & Environment, Northumbria University, Newcastle upon Tyne, NE1 8ST, UK

[4]State Key Laboratory of Solidification Processing, Shaanxi Key Laboratory of Fiber Reinforced Light Composite Materials, Northwestern Polytechnical University, Xi'an 710072, P.R. China

***Corresponding authors:** E-mail: Richard.fu@northumbria.ac.uk; heyang@nwpu.edu.cn

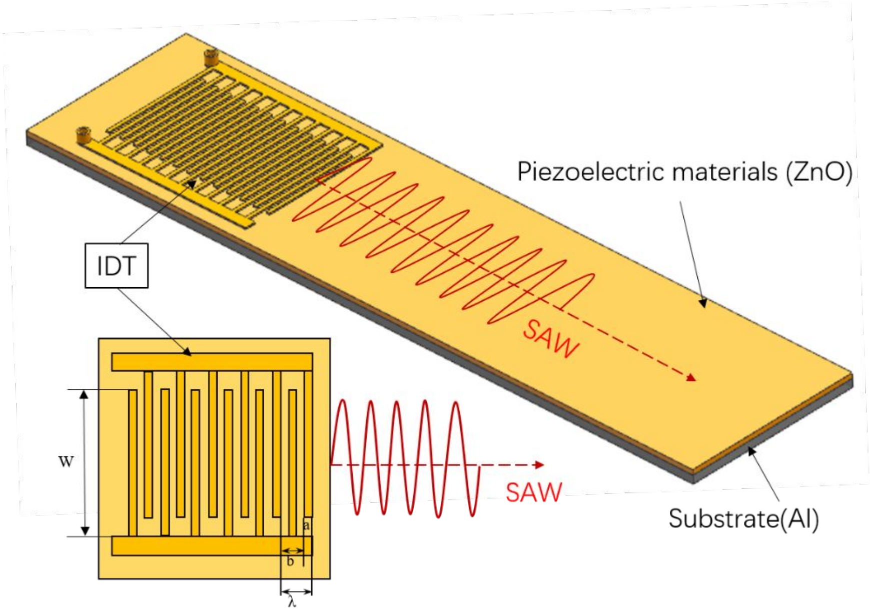

Figure S1. The SAW device prepared in this work consists of three distinct phases, i.e., a substrate of aluminum or silicon, a piezoelectric film of ZnO, and interdigital transducers, made of Al.

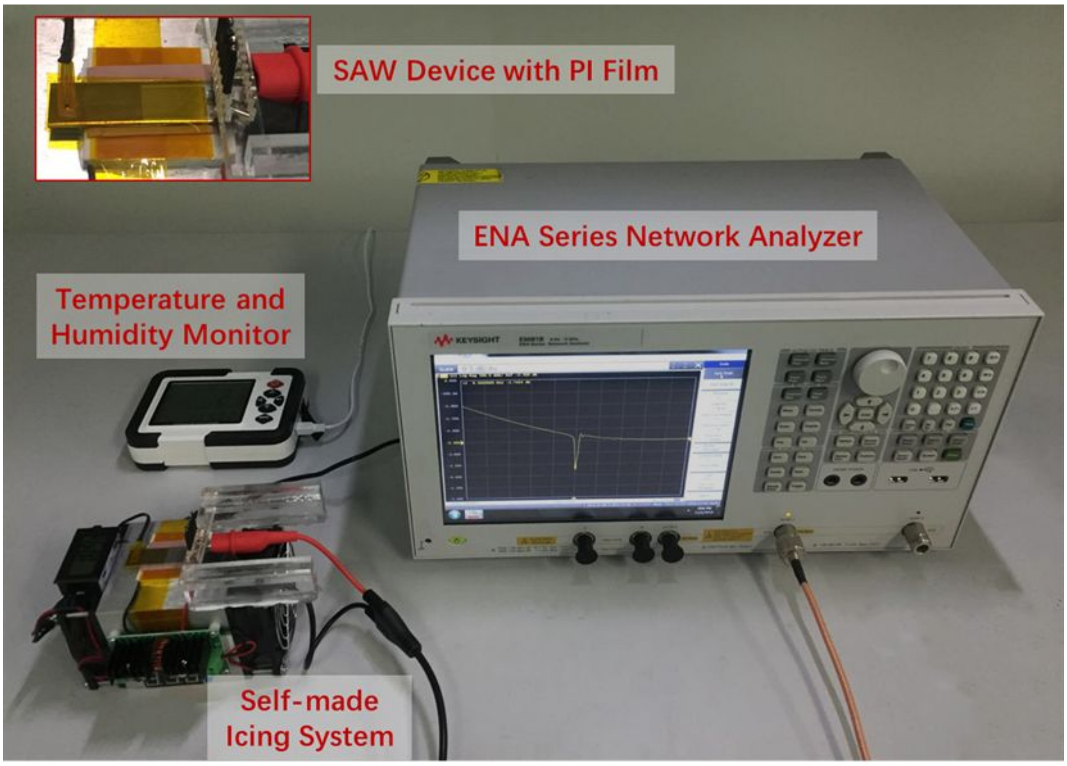

Figure S2. The reflection spectrum (S11) of the acoustic wave device was detected using a

network analyzer (Agilent E5061B)

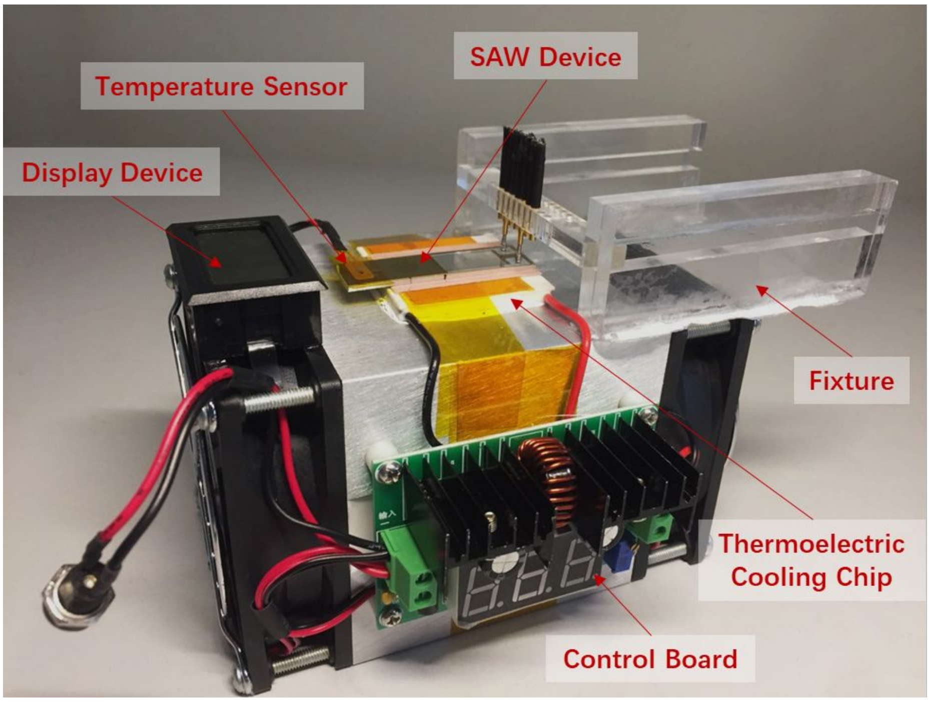

Figure S3. The SAW device was placed on a semiconductor cooler to control temperature. In an open environment without purposely controlling the humidity levels**,** the SAW device was placed on a semiconductor cooler to control temperature and the growth of the ice layer.

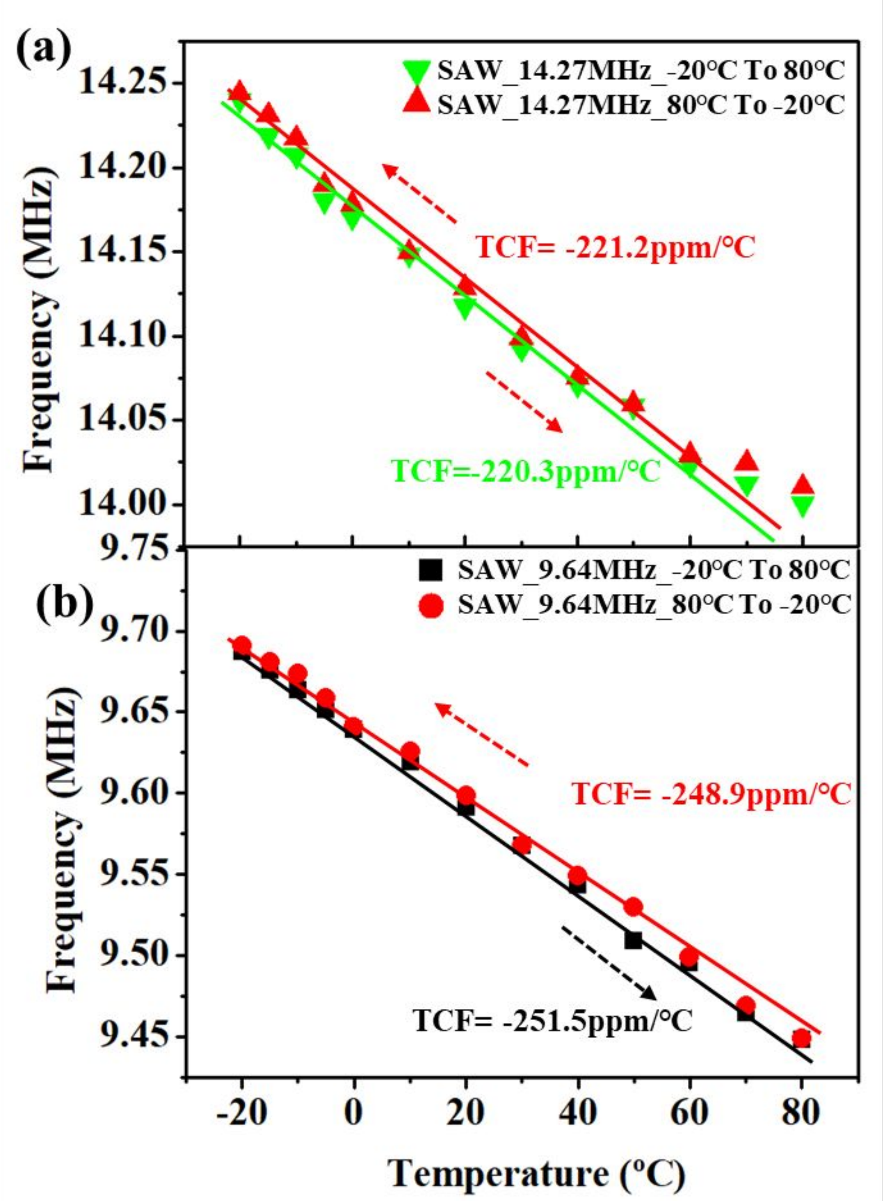

Figure S4. (a) The plots of frequency and temperature for the SAW device with λ = 200 μm and f_0 = 14.27 MHz in dry testing experiments; (b) The plots of frequency and temperature for the SAW device with λ = 300 μm and f_0 = 9.64 MHz in dry testing experiments.

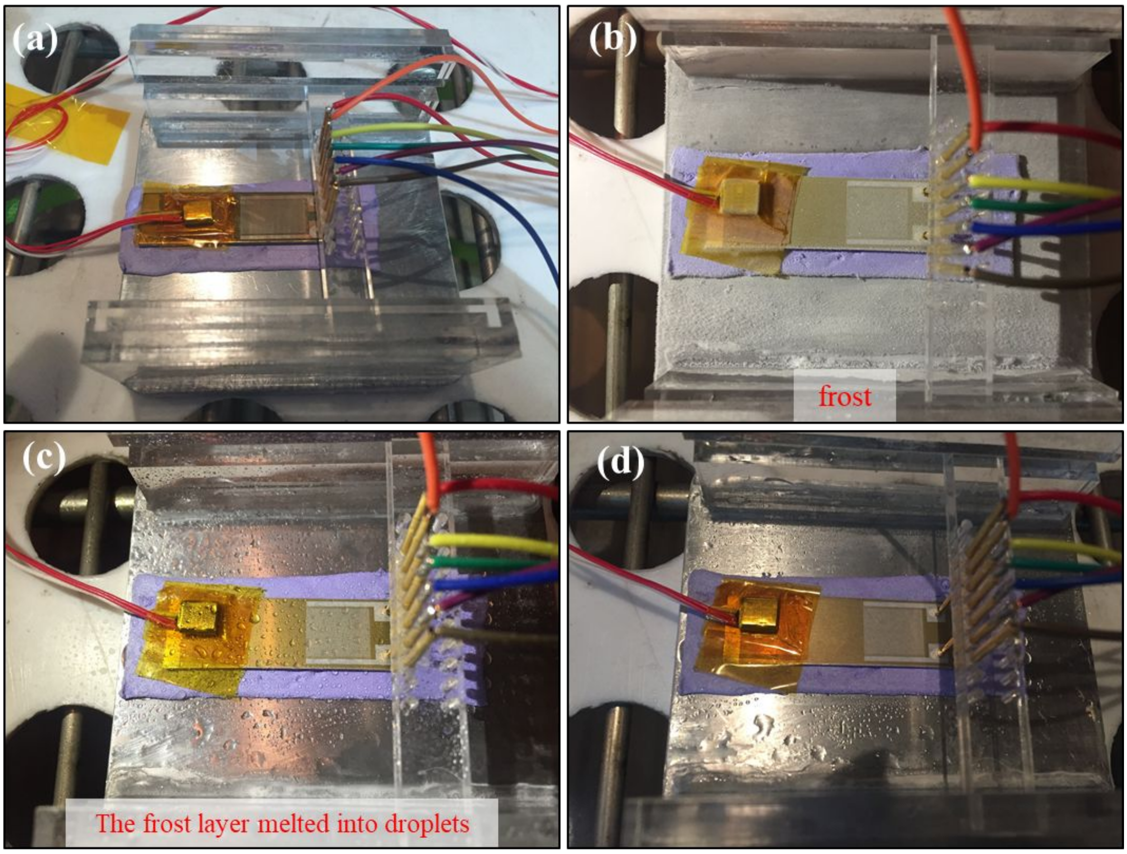

Figure S5. Photograph of covered surface of SAW device with $\lambda = 300$ µm and $f_0 = 9.64$ MHz. (a) No apparent ice formation on the surface of SAW device in a dry cooling experiment; (b) Photographs of the device's surface covered with frost; (c) The frost melted into droplets; (d) The dried device's surface.
